# The mental health of unemployed Brussels youth: the role of social and material resources

**DOI:** 10.1186/s13690-017-0187-7

**Published:** 2017-04-24

**Authors:** Kelly Huegaerts, Vanessa Puig-Barrachina, Christophe Vanroelen

**Affiliations:** 1 Interface Demography, Vrije Universiteit Brussel, Pleinlaan 5, 1050 Brussels, Belgium; 2 Barcelona Public Health Agency, Pl. Lesseps 1, 08023 Barcelona, Spain; 3 Health Inequalities Research Group (GREDS), Universitat Pompeu Fabra, Plaça de la Mercè 10-12, 08002 Barcelona, Spain

**Keywords:** Youth unemployment, Mental health, Transitions in youth

## Abstract

**Background:**

In the aftermath of the 2008 Great Recession, youth unemployment rates in the Brussels Capital Region (BCR) increased. The aim of this study is firstly to investigate the evolution of the mental health gap between employed and unemployed youth and secondly to examine the association of material and social resources with mental health of youth entering the labour market in the BCR.

**Methods:**

Two data sources are used to answer the research questions: the Belgian Health Interview Survey (HIS) data (1997 to 2013; 18- to 29-year-olds; *N* = 5,562), and the authors’ own primary data collection among Brussels youth in the transition from education to employment (2015; 18- to 29-year-olds; *N* = 1,151; BCR-sample). Prevalence ratios, bivariate and multivariate logistic regression analyses are used to explore mental distress and possible mental disorder amongst this particular group of youth.

**Results:**

The results show a consistent tendency towards increasing mental health problems for unemployed, compared to employed youth in the 1997–2013 period in the three Belgian Regions. Both social support and the living arrangements of men are related to mental distress and a possible mental disorder. The perception of a poor financial situation is related to a possible mental disorder. Our study also found that escape-avoidance behaviour is important in explaining both adverse mental health outcomes amongst women.

**Conclusions:**

This study suggests that the mental health gap between employed and unemployed youth increases and demonstrates the importance of material and social resources for the mental health of unemployed youth. These results can contribute to discussions on unemployment policies targeting vulnerable youth.

**Electronic supplementary material:**

The online version of this article (doi:10.1186/s13690-017-0187-7) contains supplementary material, which is available to authorized users.

## Background

In the aftermath of the Great Recession, many European countries were confronted with an increase in their unemployment rates [1, 2]. Compared to adults, youth was continuously and disproportionately hit by this crisis [2–4]. Ever since the start of the Great Recession in 2008 the average unemployment rate (until the second quarter of 2016) was higher for youth in the Brussels Capital Region (36.4%) than for youth in Flanders (14.3%) and Wallonia (29.3%) [5]. The BCR can be denoted as a region with a predominantly tertiary oriented labour market demand, related to its role as the capital of Belgium and Europe, and as the home town of many international institutions and headquarters. This labour market demand is contradictory to the high unemployment, which affects its inhabitants. An important explanation for this phenomenon is the educational mismatch between labour supply and demand within the region. Over 60% of the Brussels population is lower educated, which excludes them from a vast amount of the employment offered [6]. A large share of the employment is thus filled in by employees commuting from the Flemish and Walloon region [7]. This structural contradiction is known as the “Brussels paradox” [8].

Already in the classical works of Jahoda [9] and Warr^1^ [10] it was shown that the association between unemployment and mental health can be explained through a wide range of material and immaterial factors, encompassing both selection and causation mechanisms [11–18]. Parallel with the transition from education to employment, youth are confronted with several other important life changes (e.g., moving out of the parental house; first marriage or (pre-marital) break-up; having a child) that interact with entering the labour market [19]. In that context, unemployment experiences may be particularly detrimental for mental health, while at the same time mental health is an important factor in achieving labour market success [20].

Relations between (youth) unemployment and mental health are codetermined by important societal changes. The negative effects on mental health are more serious for unemployment occurring during an economic crisis [18, 21–24]. Some studies argue that structural unemployment positively affects feelings of stigmatization, shame [25, 26] and stress [27]. Negative mental health effects can also be offset by welfare state protection and other policy measures [22, 28–30]. In that regard, it should be noted that in Belgium, the right to unemployment benefits became a lot stricter from 2012 onwards, with less generous and accessible unemployment benefits for youth [31, 32]. Finally, since the 90ies also an evolution towards ‘more active labour market policies’ with an increasing focus on coaching, training and (subsidized) first work experiences has been seen [32]. Each of these macro-social and policy changes might have affected the mental health levels of unemployed youth over time. Therefore, a first objective of this study is to investigate the evolution of the ‘mental health gap’ between employed and unemployed youth in Belgium, and in Brussels in particular.

Research on the mental health effects of unemployment has often pointed towards the mediating effects of ‘contextual resources’. This concerns material resources in the first place: financial strain is found to be related to a higher risk of psychological distress and adverse wellbeing [33]. Reversely, experiencing a comfortable financial situation attenuates the mental health effects of unemployment [34]. Generous welfare state protection during unemployment can be seen as a very important source of ‘health-protecting material security’ [22, 28, 35]. However, also living arrangements are important: unemployed people living alone or with acquaintances have more adverse mental health outcomes compared to unemployed people living with a partner [36, 37]. Particularly for unemployed youth, living with one’s parents was found to be a protection against material deprivation [37]. The social resources are important as well. Low parental support increases the vulnerability for adverse mental health amongst the unemployed [37, 38], while high social support can reduce psychological distress [39]. A second objective of this study is to investigate the role of material and social resources in relation to mental health in a sample of unemployed young people at the moment of transition from education to the labour market.

Both the prevalence and type of mental health problems are different in men and women [40] and the same goes for mental health outcomes and coping styles during unemployment [41]. It is generally assumed that the effect of unemployment on mental health is less strong in women, given the different positions and roles they occupy in society and the family [42]. Moreover, while women tend to internalize social stressors (e.g., higher levels of psychological distress, higher rates of mood and anxiety disorders), men show externalising behaviour (e.g., use of medication, alcohol and drugs) [40, 43]. Research showed that unemployment correlated with a higher alcohol consumption [44] and a higher frequency of substance use [44, 45], especially amongst men [40]. Therefore, gender will be an important stratifying variable in our analyses.

Finally, the unemployment experience tends to vary with human capital [16, 46]. Recent research on the matter showed that low education is related to higher rates of psychological distress and mental disorders [40, 47]. In part this is related to the fact that the probability of exiting unemployment is higher for those with post-secondary education [48] and that - reversely - early-school-leavers have a higher likelihood of becoming unemployed [49].

In sum, this paper aims to explore the association between unemployment and mental health in Belgian youth, while paying particular attention to the BCR. This aim is translated into two sub-objectives. (1) To describe the evolution (1997–2013) of the difference in mental distress and possible mental disorder between unemployed and employed youth in the three Belgian Regions. This analysis is based on data from five waves of the Belgian Health Interview Survey (HIS). (2) To make an explanatory analysis of the determinants of mental health in a sample of unemployed Brussels youth entering the labour market. Hereby, specific attention will be paid to the effects of material and social resources. This analysis is based on a large sample of unemployed Brussels school leavers from 2015. Given the specificity of this sample, our results can provide more insight in the mechanisms linking unemployment to mental health in this specific transitional point in the life of youth.

## Methods

### Data and study population

For the first objective, data from a sub-sample of employed and unemployed youth (18- to 29-year-olds) in the five waves (1997, 2001, 2004, 2008, 2013) of the HIS were used (see Table 1). Further on, this sample will be referred to as the ‘HIS-sample’.

**Table 1:** Frequency of employed and unemployed 18- to 29-year-olds in the different Belgian regions (HIS-sample)

	Flanders		Brussels Capital Region		Wallonia	
Year	Employed	Unemployed	Employed	Unemployed	Employed	Unemployed
1997	370	58	204	66	288	88
2001	404	28	200	35	357	73
2004	373	34	214	61	315	85
2008	278	26	235	64	263	84
2013	208	21	208	63	279	72

For the second objective, information from a primary data collection conducted between August and December 2015 was used. A self-administered questionnaire was distributed at an information session for unemployed youth in Brussels (18- to 29-year-olds) entering the labour market. This informative session is organised by the Public Employment Service of the BCR, Actiris, and is a compulsory first step for all first-time enrolling youth in order to open the right to claim unemployment benefits after a waiting period of 12 months. In total, 36.77% of all eligible respondents - during the time frame of our data collection - participated in the study (*N* = 1,151), see Table 2. We have no indications of strong sampling biases. It should also be noted that, even though enrolment is related to the right to unemployment benefits, some youth are reluctant to register with Actiris. These so-called NEET-youth are not captured in our sample. Further on, this sample will be referred to as the ‘BCR-sample’.

**Table 2:** Frequency of the different categories of the independent variables and prevalence of mental distress and disorder within these categories (BCR-sample)

	Men			Women		
Variables	N	Mental distress (%)	Mental disorder (%)	N	Mental distress (%)	Mental disorder (%)
*TOTAL BCR-SAMPLE*	*501*	52.30	32.30	*605*	68.60	47.80
*Social resources*						
*Social support*	*487*	***	***	*590*	**	***
Low social support	43	58.10	39.50	28	85.70	71.40
Medium social support	61	77.00	57.40	77	84.40	67.50
High social support	383	48.80	28.20	485	65.80	43.90
*Material resources*						
*Self-perceived financial situation*	*486*	*	*	*589*	*	**
Comfortable	220	46.40	27.30	259	63.30	40.50
Modest	229	56.30	34.50	286	72.40	52.40
Poor	37	62.20	45.90	44	81.80	68.20
*Living arrangements (respondent lives…)*	*494*			*602*	*	
with his/her parents	371	49.60	29.60	401	65.10	44.90
with his/her partner	30	66.70	50.00	91	70.30	47.30
alone	42	57.10	33.30	52	82.70	61.50
with friends or acquaintances	51	56.90	39.20	58	75.90	55.20
*Educational attainment*	*490*			*592*		
Secondary education	327	51.10	33.90	322	65.20	46.30
Post-secondary education	163	53.40	28.20	270	72.60	49.30
*Escape-avoidance behaviour*						
*Used medication in the last 30 days?*	*477*	*	**	*598*	**	*
No	353	49.60	29.50	330	63.90	43.60
Yes	124	62.10	42.70	268	74.30	53.40
*Used alcohol in the last 30 days?*	*473*			*594*	***	**
No	194	49.50	33.50	320	62.20	42.80
Yes	279	55.90	33.00	274	75.50	53.60
*Used cannabis in the last 30 days?*	*467*	*		*587*	***	*
No	360	49.20	29.70	526	66.20	46.20
Yes	107	62.60	39.30	61	88.50	62.30

Chi^2^ test of significance: *** *p*. ≤ 0.000; ** *p*. ≤ 0.01; * *p*. ≤ 0.05

### Indicators

#### Independent variables

##### Employment status

In the HIS-sample, the employed are defined by the questions: “Do you have a paid job at this moment?” and “What is your current non-employment status?”. The employed were defined as those respondents contesting affirmatively on the first question; the unemployed were defined as those answering “no” on the first question, and stating to be “unemployed” in the second question. In Table 1 the number of employed and unemployed 18- to 29-year-olds in the different Belgian regions is shown for each HIS-wave.

##### Social support

In the BCR-sample, social support was conceptualised by means of the Duke-UNC Functional Social Support Questionnaire. This is an 8-item questionnaire (Cronbach’s alpha = 0.897) on a 5-point Likert scale: (1) Much less than I would like; (2) Less than I would like; (3) Some, but would like more; (4) Almost as I would like and (5) As much as I would like”. The original items were recoded into a sum scale distinguishing low (1 to 2), medium (3) and high (4 to 5) self-perceived social support (see Table 2).

*Material resources* consists of two variables in the BCR-sample. Self-perceived financial situation was included as a single item with the following response categories: (1) comfortably; (2) modest and (3) poor. Living arrangements were represented by a single variable with four categories: (1) respondent lives with his/her parents; (2) respondent lives with his/her partner; (3) respondent lives alone or (4) respondent lives with friends or acquaintances (see Table 2).

*Covariates* included in the analysis of the BCR-sample are educational level (secondary and post-secondary education) and escape-avoidance behaviour (usage of medication, cannabis and alcohol in the last 30 days). Sex is included as a stratification variable in the analyses of the BCR-sample. Because of low numbers of observations, this was not possible in the HIS-data. All indicators are further described in Table 2.

An explorative analysis showed no significant effect of age, religion or educational level of the parents nor an interaction with educational level on both mental health outcomes, which is why these covariates were not included in the final model.

#### Outcome

Mental health was measured - in both samples - using the 12-item General Health Questionnaire (GHQ12) developed by Goldberg and Williams (Cronbach’s alpha = 0.865) [50]. The GHQ12 is considered to be a good indicator for adverse mental health outcomes [51]. The 12 items with a four point Likert-scale were recoded as follows: response values 1–2 and 3–4 of each item were recoded into respectively ‘0’ and ‘1’ and subsequently summed into a scale ranging between 0 and 12. Based on this scale, two mental health outcomes were constructed using cut-off points 2+ and 4+, respectively representing “mental distress” and “a possible mental disorder” [52].

### Statistical methodology

To describe the HIS-data, the number of employed and unemployed youth are presented per region and per year (Table 1). To describe the BCR-data, the prevalence of mental distress and a possible mental disorder is presented, with corresponding Chi-squared statistics for significance testing (Table 2). To compare the prevalence of adverse mental health outcomes (objective 1) for unemployed versus employed youth over time and region, prevalence ratios (PR’s) with 95% confidence intervals (CI’s) are given (Table 3).

**Table 3:** Frequencies (N), prevalence (%) and prevalence rates (PR) and 95% confidence intervals (CI) of mental distress and a possible mental disorder in unemployed compared to employed youth in Belgium and the BCR from 1997 to 2013 (HIS-sample)

	Brussels Capital Region					Belgium				
	*Unemployed*		*Employed*		*Unemployed/ Employed*	*Unemployed*		*Employed*		*Unemployed/ Employed*
Mental distress										
*Year*	*N*	*%*	*N*	*%*	*PR (CI)*	*N*	*%*	*N*	*%*	*PR (CI)*
1997	31	50.00	69	36.13	1.38 (1.01–1.89)	85	42.08	253	30.12	1.40 (1.15–1.69)
2001	9	33.33	56	31.46	1.06 (0.60–1.88)	42	34.71	258	28.70	1.21 (0.93–1.58)
2004	22	44.00	49	29.70	1.48 (1.00–2.19)	67	43.51	207	26.20	1.66 (1.34–2.06)
2008	18	40.91	56	32.18	1.27 (0.84–1.80)	63	43.45	172	26.96	1.61 (1.29–2.02)
2013	8	57.14	36	30.00	1.90 (1.12–3.23)	38	46.91	127	27.97	1.68 (1.27–2.21)
Possible mental disorder										
*Year*	*N*	*%*	*N*	*%*	*PR (CI)*	*N*	*%*	*N*	*%*	*PR (CI)*
1997	18	29.03	32	16.75	1.73 (1.05–2.86)	50	24.75	121	14.40	1.72 (1.28–2.30)
2001	7	25.93	29	16.29	1.59 (0.78–3.27)	29	23.97	126	14.02	1.71 (1.20–2.44)
2004	11	22.00	27	16.36	1.34 (0.72–2.51)	33	21.43	100	12.66	1.69 (1.19–2.41)
2008	11	25.00	31	17.82	1.40 (0.77–2.56)	43	29.66	77	12.07	2.46 (1.77–3.41)
2013	5	35.71	14	11.67	3.06 (1.30–7.22)	22	27.16	54	11.89	2.28 (1.48–3.53)

PR (CI) in bold equals significant according to the *p* < 0.05 threshold

In order to investigate the role of social and material resources (objective 2), binary logistic regression models - stratified by gender - were fitted. Crude Odds Ratios (ORr) and 95% CI’s are presented for each covariate (model 1). Next, a multivariate logistic model was estimated, simultaneously including the social and material resources variables (model 2). A final multivariate logistic model (model 3) adds a number of relevant covariates to model 2, namely educational level and escape-avoidance behaviour. The multivariate models are presented in terms of Adjusted Odds Ratios (ORa) and 95% CI’s. Models were evaluated based on the Hosmer and Lemeshow’s Goodness of fit statistics and considered satisfactory. The data was entered, sorted, cleaned and analysed using SPSS version 23.

### Ethical considerations

Ethical approval for the BCR-study was received from the Vrije Universiteit Brussel Medical ethics board (2015/229; B.U.N. 143201525066). Participants were fully informed about the study by a researcher and the informed consent form they signed prior to their participation.

## Results

### Evolution of the mental health gap between unemployed and employed youth in Belgium

In Table 3, the prevalence ratios of mental distress and a possible mental disorder are presented for unemployed compared to employed youth in the different HIS-waves between 1997 and 2013. We found a consistent tendency towards higher PR’s for unemployed versus employed youth. For Belgium as a whole, most of these prevalence ratios show significantly higher mental distress and possible mental disorder among the unemployed. Similar observations are made for the separate regions, but due to low sample sizes the PR’s often remain insignificant (see Additional file 1 for Flanders and Wallonia). Moreover, the analyses reported in Table 3 present a pattern towards rising PR’s over the 1997–2013 period. However, a Cochrane’s Q test did not indicate that the prevalence rates of mental distress or possible mental disorder of unemployed versus employed were significantly different between the years (results not shown). Looking at the absolute prevalence it becomes clear that changing prevalence rates over years are due both to decreases in mental distress and disorder among employed youth and increases among unemployed youth.

### Relation between social and material resources and adverse mental health outcomes

In this section, we use the BCR-sample of unemployed youth in Brussels who just entered the labour market to explain part of the variation in mental health problems, by focussing on the explanatory role of social and material resources in particular.

In Table 2, descriptive analyses derived from the BCR-sample are reported. The overall prevalence of mental distress (61.3%) and possible mental disorder (40.9%) is high. The prevalence for both outcomes is higher in women then in men. Moreover, both in men and women, the prevalence of mental distress and mental disorder significantly differs according to social support, self-perceived financial situation and the use of medication in the last 30 days. The other covariates and control variables do not show a clear pattern in relation to the outcomes, with the magnitude of the association often varying alongside gender lines (see Table 2). Finally, there is no significant association between the mental health outcomes and educational attainment.

#### Mental distress

In the bivariate logistic regression analysis (Table 4, model 1), we see that male and female unemployed youth with medium social support have higher odds of mental distress compared to respondents with high social support. The same pattern holds for women with low social support, but not for men. Both men and women in a modest financial situation have higher odds compared to respondents who reported a comfortable financial situation. For women, the odds are also higher (ORr: 2.61) when indicating a poor financial situation. Women who live alone have significantly higher odds compared to women living with their parents (ORr: 2.56). The relationship with educational attainment is only borderline significant for women, showing slightly higher odds for the post-secondary educated. For both men and women, the odds in case of using medication and cannabis in the last 30 days are significantly higher compared to non-users. For the use of alcohol this is only important for women (ORr: 1.88). In the multivariate models (2 and 3), social support remains the most important explanatory factor. Material resources loses its significance, except for men who live with their partner (ORa: 2.45). The higher odds for mental distress among post-secondary educated women is significant in this model (ORa: 1.56). The use of medication and drugs amongst women is associated with higher odds of mental distress.

**Table 4:** Associations of social and material resources with mental health outcomes, before and after controlling for potential confounders. BCR-sample; odds ratio’s (OR) and 95% confidence intervals (CI)

	*Mental distress*						*Possible mental disorder*					
	Men			Women			Men			Women		
	Model 1	Model 2	Model 3	Model 1	Model 2	Model 3	Model 1	Model 2	Model 3	Model 1	Model 2	Model 3
	ORr (CI)	ORa (CI)	ORa (CI)	ORr (CI)	ORa (CI)	ORa (CI)	ORr (CI)	ORa (CI)	ORa (CI)	ORr (CI)	ORa (CI)	ORa (CI)
*Social support*												
Social resources												
High social support (ref.)	1	1	1	1	1	1	1	1	1	1	1	1
Medium social support	3.52 (1.88–6.60) ***	4.17 (2.16–8.03) ***	4.78 (2.28–10.06) ***	2.82 (1.48–5.37) **	2.41 (1.25–4.63) **	3.09 (1.54–6.21) **	3.43 (1.97–5.97) ***	4.18 (2.34–7.47) ***	4.33 (2.28–8.20) ***	2.66 (1.60–4.42) ***	2.24 (1.33–3.78) **	2.54 (1.46–4.39) **
Low social support	1.46 (0.77–2.76)	1.56 (0.79–3.08)	1.59 (0.76–3.34)	3.12 (1.07–9.15) *	2.55 (0.85–7.69)	2.65 (0.86–8.19)	1.67 (0.87–3.19)	1.87 (0.94–3.72)	2.00 (0.94–4.22)	3.19 (1.38–7.39) **	2.49 (1.04–5.95) *	3.76 (1.42–9.97) *
*Material support*												
Self-perceived financial situation												
Comfortable (ref.)	1	1	1	1	1	1	1	1	1	1	1	1
Modest	1.49 (1.03–2.17) *	1.36 (0.92–2.01)	1.39 (0.91–2.12)	1.52 (1.06–2.18) *	1.30 (0.89–1.89)	1.37 (0.91–2.05)	1.40 (0.94–2.10)	1.34 (0.88–2.05)	1.33 (0.84–2.11)	1.62 (1.15–2.27) **	1.39 (0.97–1.98)	1.52 (1.04–2.22) *
Poor	1.90 (0.93–3.89)	1.76 (0.82–3.76)	1.77 (0.80–3.90)	2.61 (1.16–5.84) *	1.73 (0.70–4.29)	2.37 (0.87–6.43)	2.27 (1.11–4.62) **	2.54 (1.18–5.49) *	2.34 (1.04–5.25) *	3.14 (1.59–6.21) **	2.04 (0.95–4.37)	2.71 (1.17–6.27) *
Family situation (respondent lives…)												
with his/her parents (ref.)	1	1	1	1	1	1	1	1	1	1	1	1
with his/her partner	2.03 (0.93–4.46)	2.32 (1.04–5.15) *	2.45 (1.03–5.82) *	1.27 (0.78–2.09)	1.23 (0.73–2.07)	1.05 (0.61–1.81)	2.37 (1.12–5.02) *	2.95 (1.36–6.37) **	4.10 (1.75–9.57) **	1.10 (0.70–1.74)	1.08 (0.67–1.74)	0.93 (0.56–1.55)
alone	1.36 (0.71–2.58)	1.16 (0.58–2.32)	1.31 (0.62–2.81)	2.56 (1.21–5.41) *	1.84 (0.81–4.20)	1.60 (0.65–3.91)	1.19 (0.60.2.34)	0.89 (0.42–1.87)	0.95 (0.43–2.13)	1.96 (1.09–3.55) *	1.48 (0.76–2.90)	1.34 (0.65–2.76)
with friends or acquaintances	1.34 (0.74–2.42)	1.36 (0.74–2.51)	1.33 (0.66–2.68)	1.69 (0.89–3.18)	1.52 (0.80–2.91)	0.84 (0.41–1.73)	1.53 (0.84–2.80)	1.55 (0.82–2.95)	2.13 (1.01–4.52) *	1.51 (0.87–2.63)	1.38 (0.78–2.44)	1.00 (0.53–1.91)
Obtained educational level												
Secondary education (ref.)	1		1	1		1	1		1	1		1
Post-secondary education	1.10 (0.75–1.60)		0.95 (0.60–1.51)	1.41 (0.99–2.01)		1.56 (1.02–2.37) *	0.77 (0.51–12.15)		0.65 (0.38–1.10)	1.13 (0.82–1.56)		1.19 (0.81–1.76)
*Escape-avoidance behaviour*												
Used medication in the last 30 days?												
No (ref.)	1		1	1		1	1		1	1		1
Yes	1.67 (1.10–2.53) *		1.44 (0.91–2.27)	1.63 (1.14–2.32) **		1.60 (1.09–2.36) *	1.79 (1.17–2.73) **		1.54 (0.96–2.48)	1.48 (1.07–2.04) *		1.52 (1.07–2.17) *
Used alcohol in the last 30 days?												
No (ref.)	1		1	1		1	1		1	1		1
Yes	1.30 (0.90–1.87)		1.20 (0.76–1.89)	1.88 (1.32–2.68) **		1.44 (0.93–2.23)	0.98 (0.66–1.44)		0.85 (0.51–1.41)	1.55 (1.12–2.14) **		1.51 (1.00–2.27) *
Used cannabis in the last 30 days?												
No (ref.)	1		1	1		1	1		1	1		1
Yes	1.73 (1.11–2.70) *		1.50 (0.90–2.48)	3.95 (1.76–8.85) **		2.59 (1.07–6.25) *	1.53 (0.98–2.39)		1.62 (0.95–2.75)	1.92 (1.12–3.32) *		1.21 (0.64–2.31)

*** *p*. ≤ 0.000; ** *p*. ≤ 0.01; * *p*. ≤ 0.05

#### A possible mental disorder

The bivariate logistic regressions (Table 4, model 1) show that for both men and women the odds of a possible mental disorder are higher when having medium social support compared to high social support. Additionally, having low social support showed higher odds (ORr: 3.19) amongst women. For both men and women, the odds are higher when living in a financially poor situation compared to living in a comfortable financial situation. The same relation can be found amongst women in households with a modest financial situation. Male respondents who live with their partner, have significantly higher odds (ORr: 2.37) to report a possible mental disorder. For women, we found a significant association (ORr: 1.96) for respondents living alone. The use of medication also resulted in significantly higher odds amongst both men and women. The use of alcohol and drugs appears to be only significant amongst women (ORr: 1.55 and 1.92).

In the 2^nd^ and 3^rd^ model, the contribution of medium social support remains important for men and women, while low social support only shows significantly higher odds for women (ORa: 2.49 and 3.76). A poor financial situation remains relevant among men (ORa: 2.54 and 2.34). For women, the financial situation is only relevant in the third model (ORa modest: 1.52; ORa poor: 2.71). The living arrangements are no longer significant for women. For men living with friends or acquaintances (ORa: 2.13) and living with a partner (ORa: 4.10) is significant in explaining a possible mental disorder for men. Women’s escape avoidance behaviour remains significant except for the use of cannabis. For men, there is no significant effect of escape avoidance behaviour on mental distress.

## Discussion and limitations

This paper investigates the association of social and material resources with mental health amongst unemployed youth in their transition from education to employment. In the BCR-sample, we found a high prevalence of mental distress and a possible mental disorder. Compared with the prevalence found for both mental health outcomes in unemployed youth from the 2013 HIS-sample (see Table 3), the prevalence for poor mental health in the BCR-sample is very high. A potential explanation is the association between the duration of the unemployment spell and mental health, which follows a U-shaped pattern (a large initial impact, decreasing over time, but growing again with the duration of the unemployment spell) [10, 18, 53]. The youth in our sample have just entered the labour market and could be expected to experience a large initial impact. The HIS-sample, in contrast, can be assumed to be more heterogeneous with regard to the unemployment duration, since it is a mere random sample.

In line with existing literature, adverse mental health outcomes are systematically higher for unemployed compared to employed youth (objective 1). Moreover, our results suggest that the mental health gap between employed and unemployed youth increases over time. This is merely an indicative pattern, since a Cochrane’s Q test did not indicate that the prevalence rates of mental distress or possible mental disorder of unemployed versus employed were significantly different between the years. However, in some instances the absolute differences are huge: e.g., in 2001 the unemployed had a 6.01% higher prevalence of mental distress compared to the employed; in 2013 the same difference was 18.94% (data for Belgium). This makes us believe that the lack of a significant trend is mainly an artefact of low N -and that it is indeed appropriate to consider the fact that their might be an increasing mental health gap, both caused by improved mental health among the employed and deteriorated mental health among the unemployed. A first possible explanation for this evolution is related to the restrictive evolution of the welfare state protection from the 90ies onwards. We know that welfare state protection during unemployment can form a ‘health-protecting material security’ [22, 28–30, 35]. A second explanation, for the two most recent waves, could be the negative effects of the economic crisis on mental health during unemployment [18, 21–24].

Our study identified the importance of social and material resources in association with mental distress and a possible mental disorder (objective 2). The results show that social support can be considered a consistent protective factor for both adverse mental health outcomes among men and women. This corresponds with previous findings about the protective role of social support in the case of mental health [37–39, 54]. We also found that, for both men and women, a poor financial situation was related to a higher likelihood of a possible mental disorder. This finding is supported by the literature, which found positive associations between mental health and a comfortable financial situation in youth [33, 34, 54]. We also found that living with a partner is consistently related to both adverse mental health outcomes for men, which is consistent with the literature as well [36]. Men in the BCR-sample who lived with a partner were on average 24 years old. A possible explanation for the association could be the presence of the male breadwinner perspective amongst this group.

Previous literature showed that escape-avoidance behaviour is more common amongst men [40, 44, 45]. Contrary to this, our results show that escape-avoidance behaviour is only significant in explaining mental health amongst women. Even though escape-avoidance behaviour is believed to be a predominantly male phenomenon, research found that the use of medication is more likely among women [55] and suggest that the “sex gap” for alcohol and drug use has been narrowing over time [55–57]. Other literature suggests that men show escape-avoidance behaviour to increase positive feelings, whereas women will do this to decrease negative feelings [58].

This study has a number of limitations. Within this research field there is a clear on-going debate about the direction of the relation between unemployment and mental health (selection versus causation) [13–17]. Based on this cross-sectional data no statements about causality can be made. As also stated by Leach et al. [59], cross-sectional data does not necessarily reflect the start of mental health problems. Readers should be aware of that when interpreting our results. A second limitation relates to potentially relevant information that was not available in the survey. Due to constraints of questionnaire length, we have limited information on some variables such as the specific types of medication used. More extensive research on these particularities is thus highly recommended. A third limitation that we need to take into account is linked to the retrospective nature of some of the questions in the BCR-questionnaire, which may have caused recall bias. To minimize recall bias, the presence of this type of questions was limited. To minimize bias related to the comprehension of the questionnaire at a semantic level, a pre-testing was organised to generate the questionnaire.

Despite these limitations, our findings can be considered as highly relevant with regard to the understanding of youth unemployment in urban regions. Our results highlight the association of unemployment and mental health, as well as the explanatory impact of material and immaterial resources in this association. These findings can contribute to the debate on how to guide this specific group of youth in their transition from education to employment. Youth can benefit from a multidisciplinary guidance approach, which helps them not only to find a job, but also to e.g., establish financial security and find affordable living arrangements. Our findings can also contribute to the policy debate on granted social benefits as recently done by Vahid Shahidi et al. [35]. This study found that the government can mitigate unemployment-related health inequalities by expanding the generosity and scope of social protection policies. Despite these strengths and contributions, more research on the topic is recommended.

## Conclusions

The aim of this paper was to (1) describe the difference in mental health outcomes between unemployed and employed youth over time and region and (2) to explore the association of social and material resources with mental health amongst unemployed youth entering the labour market in the Brussels Capital Region.

This study found a high prevalence of mental distress and possible mental disorder among Brussels youth in the transition from education to employment. Secondly, when comparing unemployed with employed youth our results suggest that the mental health gap between both has been increasing. Thirdly, our study found that the role of social support is associated with both mental distress and a possible mental disorder for men and women. Living arrangements is an important factor for men and escape-avoidance behaviour is an important factor for women. The association of the financial situation is only important in understanding a possible mental disorder for both men and women.

In short, our results suggest the worsening of the mental health situation of unemployed youth compared with that of employed youth, as well as the importance of social and material resources in explaining the mental health situation of unemployed youth. By doing this, our study fills an existing gap in the research framed within the Great Recession.

## Additional file

Additional file 1: Included in this file is a table showing additional data on PR’s and 95% CI’s of mental distress and a possible mental disorder in unemployed compared to employed youth in Flanders and Wallonia from 1997 to 2013 using the HIS-sample. (PDF 32 kb)

## Abbreviations

BCR: Brussels Capital Region
CI: Confidence interval
GHQ: General Health Questionnaire
HIS: Health Interview Survey
ORa: Adjusted Odds Ratio
ORr: Crude Odds Ratio
PR: Prevalence ratio

## Footnotes

1 The Vitamin theory developed by Warr draws upon the Latent Deprivation Theory of Jahoda [10].
