# Supplementary material for: The mental health of unemployed Brussels youth: the role of social and material resources
Source: Arch Public Health. 2017 Apr 24;75:19. doi: 10.1186/s13690-017-0187-7 (PMC5402049; doi:10.1186/s13690-017-0187-7)
Supplement: Additional file 1: — Included in this file is a table showing additional data on PR’s and 95% CI’s of mental distress and a possible mental disorder in unemployed compared to employed youth in Flanders and Wallonia from 1997 to 2013 using the HIS-sample. (PDF 32 kb) [file 13690_2017_187_MOESM1_ESM.pdf]

Table 1 PR’s and 95% CI’s of mental distress and a possible mental disorder in unemployed compared to employed youth 1997-2013 in Flanders and Wallonia. HIS-sample

	**Wallonia**					**Flanders**				
	Unemployed		*Employed*			*Unemployed*		*Employed*		
**Mental distress**										
Year	*N*	*%*	*N*	*%*	*PR (CI)*	*N*	*%*	*N*	*%*	*PR (CI)*
**1997**	37	44.58	86	30.28	**1.47 (1.09-1.98)**	17	29.82	98	26.85	1.11 (0.72-1.71)
**2001**	25	37.31	112	33.84	1.10 (0.78-1.56)	8	29.63	90	23.08	1.28 (0.70-2.36)
**2004**	33	44.00	87	30.85	**1.43 (1.05-1.94)**	12	41.38	71	20.70	**2.00 (1.24-3.32)**
**2008**	36	45.57	63	29.86	**1.53 (1.11-2.10)**	9	40.91	53	20.95	**1.95 (1.12–3.41)**
**2013**	24	44.44	45	26.16	**1.70 (1.15-2.51)**	6	46.15	46	28.40	1.63 (0.86-3.07)
**Possible mental disorder**										
Year	*N*	*%*	*N*	*%*	*PR (CI)*	*N*	*%*	*N*	*%*	*PR (CI)*
**1997**	23	27.71	41	14.44	**1.92 (1.23-3.01)**	9	15.79	48	13.15	1.20 (0.62-2.31)
**2001**	15	22.39	54	16.31	1.37 (0.83-2.28)	7	25.93	43	11.03	**2.35 (1.17-4.72)**
**2004**	16	21.33	37	13.12	1.63 (0.96-2.76)	6	20.69	36	10.50	1.97 (0.91-4.29)
**2008**	25	31.65	26	12.32	**2.57 (1.58-4.17)**	7	31.82	20	7.91	**4.03 (1.92-8.46)**
**2013**	12	22.22	19	11.05	**2.01 (1.05-3.87)**	5	38.46	21	12.96	**2.97 (1.34-6.57)**

PR (CI) in bold equals significant according to the $p<0.05$ threshold
